# Flow dynamics of droplets expelled during sneezing

**DOI:** 10.1063/5.0067609

**Published:** 2021-11-02

**Authors:** Prateek Bahl, Charitha de Silva, C. Raina MacIntyre, Shovon Bhattacharjee, Abrar Ahmad Chughtai, Con Doolan

**Affiliations:** 1 School of Mechanical and Manufacturing Engineering, UNSW Sydney, Kensington NSW 2052, Australia; 2 Biosecurity Program, The Kirby Institute, UNSW Sydney, Kensington, NSW 2052, Australia; 3 School of Population Health, UNSW Sydney, Kensington, NSW 2052, Australia

## Abstract

Respiratory infections transmit through droplets and aerosols generated by the infected individual during respiratory emissions. It is essential to study the flow dynamics of these emissions to develop strategies for mitigating the risk of infection. In particular, the dynamics of droplets expelled during violent exhalations such as sneezing is crucial, but has received little attention to date. Here, for the first time, we present the results of droplet dynamics of 35 sneezes, obtained from four volunteers, using particle tracking velocimetry experiments. Our results reveal a mean droplet velocity of 2–5.4 m/s across the different subjects. These values are significantly lower than what is usually assumed in the studies simulating or replicating sneezes. Furthermore, the large variation in droplet speeds, flow direction, spread angle, and head movement is also quantified. These findings will enable the refinement of models and simulations of sneezes toward improving infection control guidelines.

## I. INTRODUCTION

Infectious disease outbreaks, epidemics, and pandemics raise questions around the mode of transmission and protection from respiratory infections. Transmission of infection occurs through the transfer of pathogens from one host to another, and for respiratory infections, these pathogens are largely carried through droplets and aerosols generated by the infected individual during respiratory emissions (such as breathing, speaking, coughing, sneezing, etc.). Our understanding of respiratory disease transmission has evolved over time. In the late 19th century, Flügge^1^ investigated droplet-sprays using culture plates and concluded that the respiratory droplets are relatively large and settle quickly within few feet. In early 20th century, Wells^2^ introduced a cut-off limit of 100 μm to differentiate between large and small droplets and concluded that large droplets settle within immediate vicinity of the subject. However, recent studies challenge this large versus small droplet dichotomy and characterize these exhalations as multi-phase cloud of droplets, which involves complex interactions of fluid phase with the environment.^3,4^ Therefore, to better understand the infection transmission, it is essential to study the flow dynamics of exhalations and the expelled droplets/aerosols, in particular for violent exhalations such as sneezes, as very limited data are available over a variety of conditions and subjects which this study aims to address.

The infection control guidelines for the prevention of respiratory transmission make an assumption on the safe distance based on the extent of the spread of respiratory droplets to prevent from ‘droplet’ transmission of infection.^5,6^ These guidelines are based on the aerobiology studies from 1930s and 40s when measurement techniques were very limited (see Bahl *et al.*^6^ for a recent review). Specifically, the studies by Jennison^7^ in 1940s, which used high-speed illumination and film-based cameras to capture droplets expelled by sneezes, estimated an initial speed of droplets as ∼46 m/s and a spatial spread of 1 m from the subject.

Over the last three decades, significant advancements have been made to experimentally examine complex flow fields with the capacity to capture multi-component, multi-dimensional spatial data.^8^ Of these techniques, image-based methods such as Particle Image/Tracking Velocimetry (PIV/PTV) have gained significant popularity and are used extensively to understand the flow dynamics of coughs. In the early 21st century, a significant amount of work has been done on the measurement of respiratory flows using image-based techniques. Zhu *et al.*^9^ used Particle Image Velocimetry (PIV) to estimate the velocity of cough airflow and found initial velocity between 6 and 22 m/s, with the average value of 11.2 m/s. However, in their study, the cough generated was not natural as wheat flour was ingested by the subjects as tracer for the visualizations. In 2009, Tang *et al.*^10^ examined the airflow of coughs in a non-evasive fashion without any tracer using schlieren imaging. They estimated an airflow velocity of 8 m/s from a single cough of a 57-year-old volunteer. Chao *et al.*^11^ used PIV on a larger sample size of 50 coughs each by two volunteers and estimated maximum airflow velocity by the male volunteer as 13.2 m/s and by female volunteer as 10.2 m/s. In 2011, Vansciver, Miller, and Hertzberg^12^ also used PIV on an even larger dataset of 195 coughs from 29 volunteers and reported maximum velocities ranging from 1.5 to 28.8 m/s. In 2013, Nishimura *et al.*^13^ used volumetric illumination and employed Particle Image Velocimetry to analyze coughs and sneezes and estimated the velocity of sneeze airflow to be greater than 6 m/s. This study was limited to only one subject. In 2013, Tang *et al.*^14^ used shadow-graph imaging and reported a maximum airflow velocity of 4.5 m/s among the sneezes from six subjects. Later in year 2016, Scharfman *et al.*^15^ captured closed range droplets expelled from sneezes using high-speed back-illuminated imaging and reported a maximum velocity of 14 m/s for droplets and 35 m/s for ligaments. There was no mention of number of subjects in this study.

In 2020, due to the COVID-19 pandemic, there was a surge of studies on respiratory flows, but most are based on either numerical modelling^16–19^ or use pressure driven simulators,^20,21^ and only few used human experiments.^22–25^ Han *et al.*^22,23^ used PIV to measure speaking, coughing, and sneezing airflow velocities. They reported a maximum airflow velocity of 6.25, 15.3, and 15.9 m/s for speaking, coughing, and sneezing, respectively.

Since most of the studies on sneezing were limited to only few sneezes, they were limited in terms of subject-to-subject variation. One of the reasons for this limited sample size is the difficulty to induce a controlled sneeze for experimental studies. Moreover, the limited attention given to sneezes could be due to the high frequency of coughing, as it is a more common symptom in certain respiratory infections.^26,27^ Despite this, it is important to study sneeze flows as a single sneeze generates significantly more droplets per event.^28^

A major limitation of most of the studies on the flow dynamics of respiratory exhalations is the lack of data on the dynamics of the droplets expelled during these exhalations. The dynamics of the droplets expelled in these multi-phase respiratory flows determines the spread and the risk limit of infection transmission, and only few experimental studies to date have focused on this aspect. Furthermore, understanding it is also essential for better mathematical modeling and to perform accurate numerical simulations. Most simulations for coughs employ a flow profile based on experiments by Gupta *et al.*,^29^ and it is also used to introduce the droplets in the flow.^18^ Accordingly, in this study, for the first time, we present the results of droplet dynamics of 35 different sneezes obtained from four healthy volunteers. A set of experiments were performed by employing light sheet illumination to capture individual droplets in a slice of sneeze flow and then performing Particle Tracking Velocimetry (PTV) on the captured high-speed image sequences.

## II. METHODS

### A. Experimental setup

**FIG. 1.**
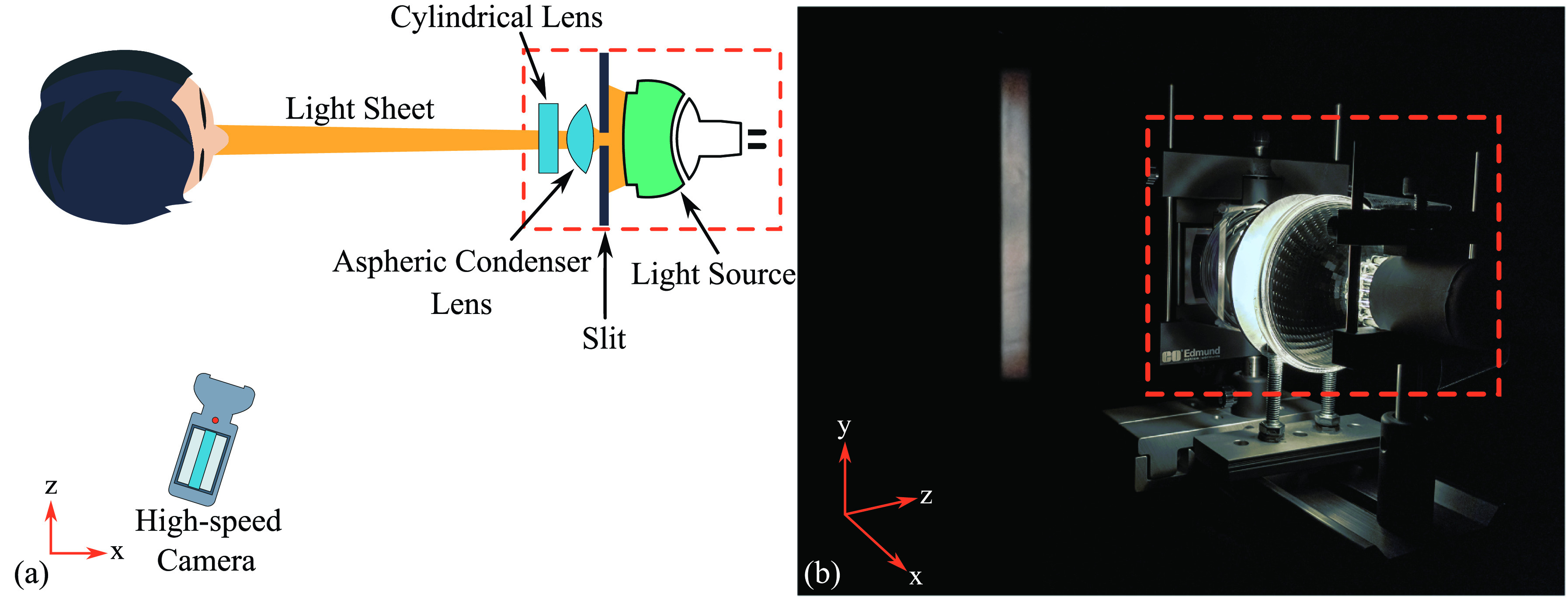
Schematic of the experimental setup used to capture the droplets expelled during sneezes (Reproduced with permission from Bahl *et al.*, “An experimental framework to capture the flow dynamics of droplets expelled by a sneeze,” Exp. Fluids. **61**, 176 (2020). Copyright 2020 Springer Nature). (a) Top view of the setup. (b) Isometric view of the setup with red rectangle depicting the light source and collimating optics.

Droplets expelled during sneezes were captured by employing a high-speed camera and light sheet illumination. Since the use of laser was not possible, light sheet illumination and high-speed motion capture were important to resolve the details of individual droplets expelled during a sneeze. Moreover, an advantage presented by light-sheet illumination is the less frequent out of plane motion of the droplets due to the sheet thickness, resulting in significantly better tracking. Accordingly, a vertical (*xy*) plane was illuminated using an LED light source (GsVitec MultiLED QT) with a beam divergence of 15° and collimating optics consisting of a 63.5×1.6 mm slit, an 80 mm aspheric condenser lens with a focal length of 59 mm, and a 50 × 50 mm plano-concave cylindrical lens with a focal length of 38 mm (see Bahl *et al.*^30^ and de Silva *et al.*^31^ for further details). This setup provided a horizontal divergence angle of 1.4° and a sheet thickness of 12±2 mm in the field of view captured. Schematic of the setup is shown in Fig. 1.

A high-speed monochrome camera (nac MEMRECAM HX-7s) was used to record videos of sneezes at a resolution of 1920 × 1080 pixels with the frame rate ranging from 1500 to 2000 frames-per-sec (fps). The camera was located at a distance of 60 cm from the subject and positioned at an angle of 80° to take the advantage of mie-scattering properties of the particles (Fig. 1). The field of view captured using the current setup is ∼600×400 mm, and the resolution obtained is ∼320 μ m/pixel. However, a limitation of this type of imaging is the ability to dimensionalize the sizing of the droplets detected, as the size on the image is a function of various parameters such as the intensity of the light available and the angle of the imaging sensor, etc.^32^

For recording sneezes, the head of the subject was positioned in front of a black backdrop facing the light source, such that the (*xy*) plane was in the middle of the face (Fig. 1). Once the head was positioned, the sneeze was induced using a tissue paper to stimulate the mucus membrane of the nasal cavity. For the experiments, an informed consent was taken from all the participants, and an approval was obtained from the University of New South Wales Human Research Ethics Committee [HC180830].

### B. Particle tracking velocimetry

Particle Tracking Velocimetry (PTV) was performed on the high-speed image sequence to compute the flow speeds of expelled droplets. Images were processed using the LaVision DaVis 8.4 software. Due to the high seeding density in certain regions, all the frames were processed using a hybrid Particle Tracking Velocimetry technique, where the procedure first performs a Particle Image Velocity (PIV) pass on the image sequence to obtain a velocity field estimate and then tracks individual particles using the PTV algorithm.^33^ The interrogation window used for PIV step starts with an initial interrogation window size of 96 × 96 pixels and final size of 48 × 48 pixels, which correspond to ∼30×30 mm and ∼15×15 mm, respectively, with an overlap of 75%. For the PTV step, a particle size ranging between 4 pixels and 50 pixels was employed, and an interrogation window of 64 pixels was used to cover the range of droplet sizes present. The hybrid approach used for particle tracking provides high accuracy with a maximum estimation error of ±0.5 pixels.^33^

### C. Image processing

In order to perform accurate particle tracking, a sequence of image processing steps were employed to process the high-speed image sequences. The background and sensor noise in the images were removed by average background subtraction. Images were further processed using temporal moving average subtraction to further reduce noise. The operation is defined by the following equations:

$$\begin{array}{c} I=I_{o}-\left[\frac{1}{n}\sum_{j=1}^{n}I_{j}\right]\\ I_{j}=\frac{1}{k}\sum_{i=p-k+1}^{p}I_{i} \end{array}.$$

Here *k* is the number of pixels used for performing moving mean of image (*I_o_*) with a vertical resolution of *p* pixels and *n* is the number of images used for temporal average of moving means (*I_j_*).

The perspective distortion that was introduced through the angled view of the camera was removed by creating and applying a transform based on a calibration target with 27 × 29 dots and a dot spacing of 5 mm. The motion of the subjects' head was tracked and stabilized using a template matching algorithm that utilized 2D normalized cross-correlation. Finally, the facial contour of the subject was detected to remove erroneous data, by creating a dynamic mask, using 2D convolution filters and a Fuzzy Inference System. All of these operations are detailed further in Bahl *et al.*^30^

## III. RESULTS AND DISCUSSION

This section presents the results obtained by applying aforementioned methodology on 35 different sneezes recorded from four subjects. The number of sneezes recorded from subjects S1, S2, S3, and S4 was 7, 6, 11, and 11, respectively. All the experiments were conducted in a temperature-controlled room with an ambient temperature of 22°C. Subjects S1 and S2 were females, and subjects S3 and S4 were males. All the four subjects were non-smoking healthy adults within the age range of 25–35 years.

**FIG. 2.**
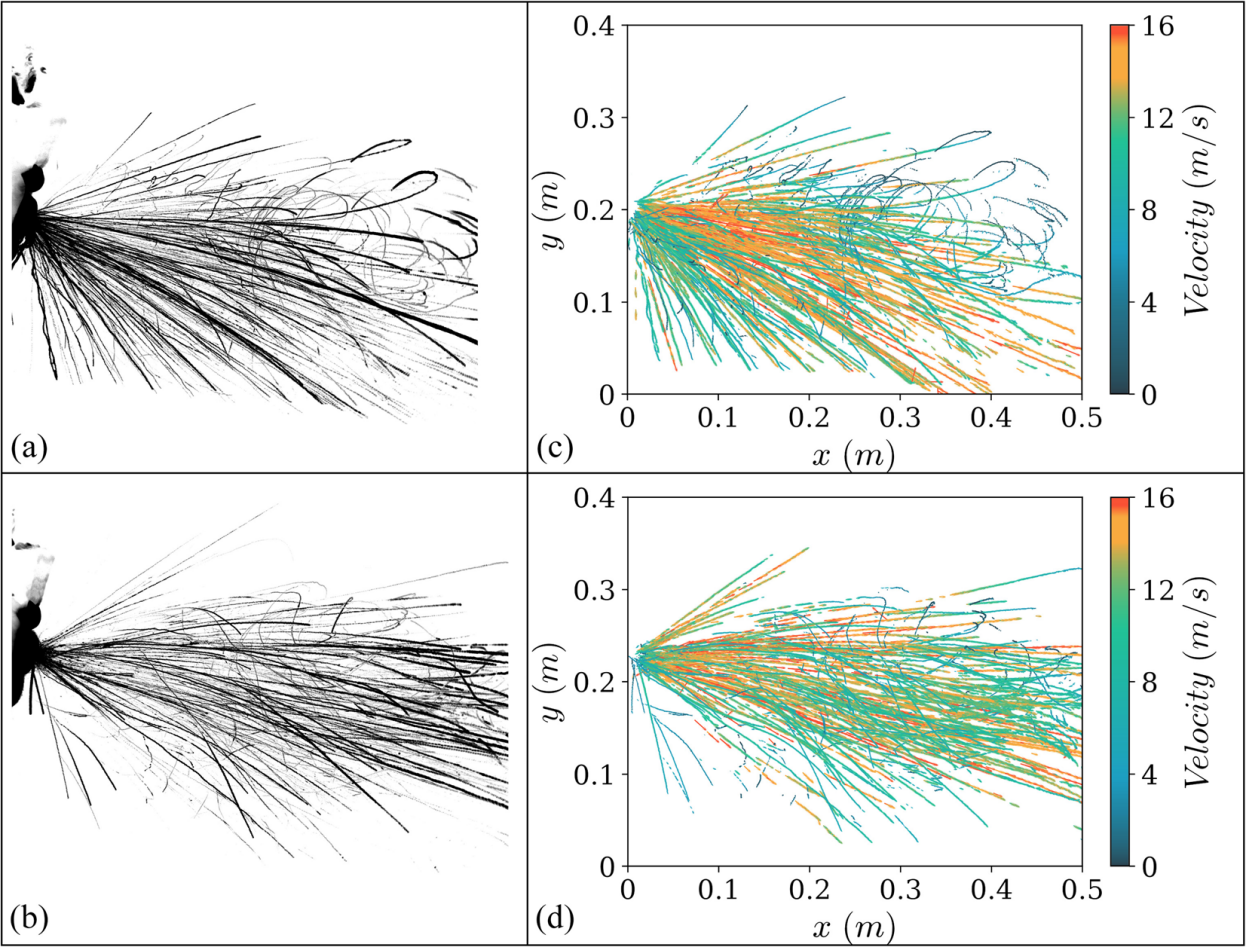
Comparison of tracks detected from high-speed frames and from Particle Tracking Velocimetry (PTV). (a) and (b) show the droplet tracks of two representative sneezes obtained by stacking the captured frames; (c) and (d) show the results using the PTV algorithm for the corresponding sneezes.

In order to visualize the accuracy of the droplet motion from the method used, Fig. 2 shows the comparison of the droplet trajectories detected for two representative sneezes. Specifically, Figs. 2(a) and 2(b) show the droplet trajectories obtained by stacking and summing the high-speed image sequence for the complete duration of two different sneezes, and Figs. 2(c) and 2(d) show the droplet tracks obtained from PTV. Most of the droplets observed within 12–16 m/s are observed to follow a ballistic trajectory and leave the field of view; however, other droplets are also observed to lose momentum rapidly leading to abrupt changes in direction. These observations can be explained by the drag force experienced by the droplets together with the evaporation and reduction in the size of droplets along the path. This leads to reduction in stokes number, resulting in the entrainment of these droplets in the turbulent puff.^3^

### A. Droplet dynamics

**FIG. 3.**
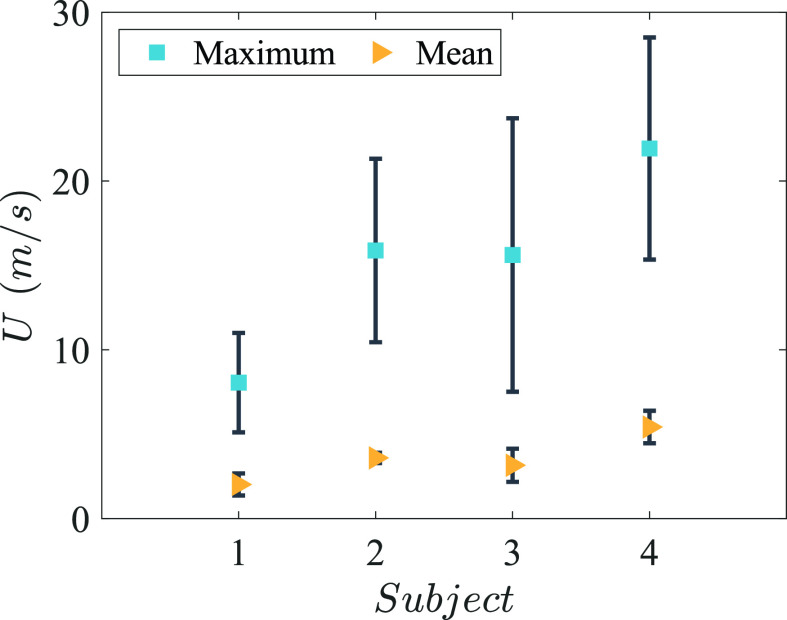
Average maximum and mean droplet velocities in 0 < *x* < 100 mm region among different sneezes for four different subjects. Error bars are the standard deviation among different sneezes from same subject.

**FIG. 4.**
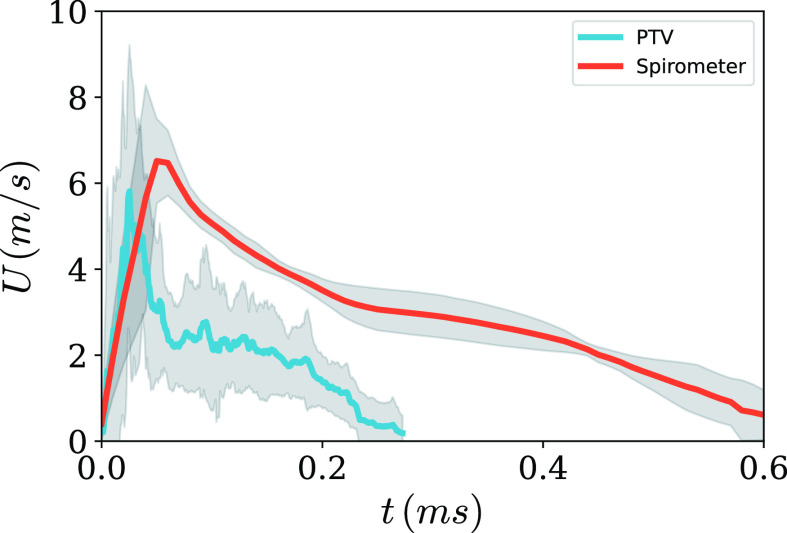
Comparison of airflow velocity (spirometer) and average droplet velocity (PTV) for subject S3. Gray-shaded region shows the standard deviation.

The maximum value of average droplet velocity (in 0<x<100 mm region), for all the subjects, ranged from 8 m/s (standard deviation (SD) = 2.9 m/s) for subject S1 to 21.9 m/s (SD = 6.5 m/s) for subject S4, as shown in Fig. 3. Figure 4 shows the comparison of airflow velocity measured with a handheld spirometer with the average droplet velocity in 0<x<100 mm region for subject S3. It can be observed that the airflow lasted for approximately 0.6 s, whereas the droplets were expelled only in the initial 0.25 s. It should be noted that the flow rate obtained from the spirometer was converted to velocity using the diameter of the spirometer mouthpiece, which was approximately 4 cm. It can also be observed that the average droplet velocity and the flow rate follow a similar trend of a sudden rise in velocity and then a gradual decline for the rest of the duration. Assuming the droplets expelled follow the airflow, for all the subjects, based on an estimated mouth diameter of approximately 30 ± 5 mm (obtained using image analysis of high-speed frames) and kinematic viscosity of $1.5\times 10^{-5}$ m^2^/s, the Reynolds number of the flow ranged from $Re=1.6\times 10^{4}$ to $Re=4.38\times 10^{4}$. For all the sneezes (n = 35), the mean value of the maximum droplet velocities observed was 16.5 m/s (SD = 7.5 m/s) with a median value of 15.8 m/s. The mean value of the average droplet velocities in 0<x<100 mm region was quite consistent among different sneezes from all the four subjects, ranging from 2 m/s (SD = 0.65 m/s) for subject S1 to 5.4 m/s (SD = 0.96 m/s) for subject S4, as shown in Fig. 3. The high variation observed in the maximum velocity among different sneezes also implies that very few droplets travel at velocities greater than 10 m/s, and since only a slice of flow was illuminated, they might not be captured in certain sneezes. Nonetheless, mean droplet velocity was quite consistent among the sneezes and is similar to the airflow velocity of 4.5 m/s observed by Tang *et al.*^14^

**FIG. 5.**
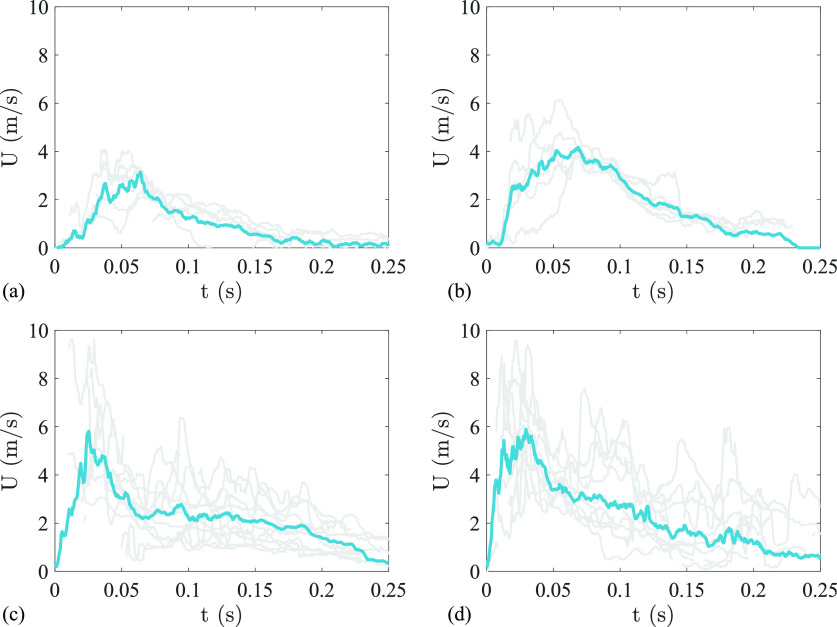
Variation in mean droplet velocities in 0 < *x* < 100 mm region, among different sneezes of (a) subject S1, (b) subject S2, (c) subject S3, and (d) subject S4. Gray plots show the data for each repeat, and blue plot shows the mean value of all the repeats from the subject.

The change in mean droplet velocities with time, in the region 0<x<100 mm, for the sneezes of all the subjects is presented in Fig. 5. The maximum values of mean droplet velocities for subjects S1, S2, S3, and S4 were 3.7, 4.3, 6.7, and 6.3 m/s, respectively. In all cases, we observe an initial rise in the velocity, which peaks around 50 ms, and then there is a gradual decrease in the velocity for the remaining duration of the sneeze. This implies that most of the fastest moving droplets are expelled at approximately 50 ms from the onset of the sneeze. This peak in droplet velocity also coincides with the peak airflow velocity measured using the handheld spirometer (see Fig. 4). For subjects S1, S2, S3, and S4, this peak was observed at 66 ± 13, 68 ± 25, 26 ± 11, and 30 ±7 ms, respectively. It can also be observed from Fig. 5 that the variation in mean velocity decreases with time for subjects S1 and S2, but there is no appreciable change in the case of subjects S3 and S4, which points toward a higher variability in the sneezes from subjects S3 and S4 as compared to S1 and S2. Furthermore, this inter- and intra-subject variation in sneezes also implies that various parameters and the associated variations should be incorporated in the modeling studies for a realistic understanding of the droplet spread.

**FIG. 6.**
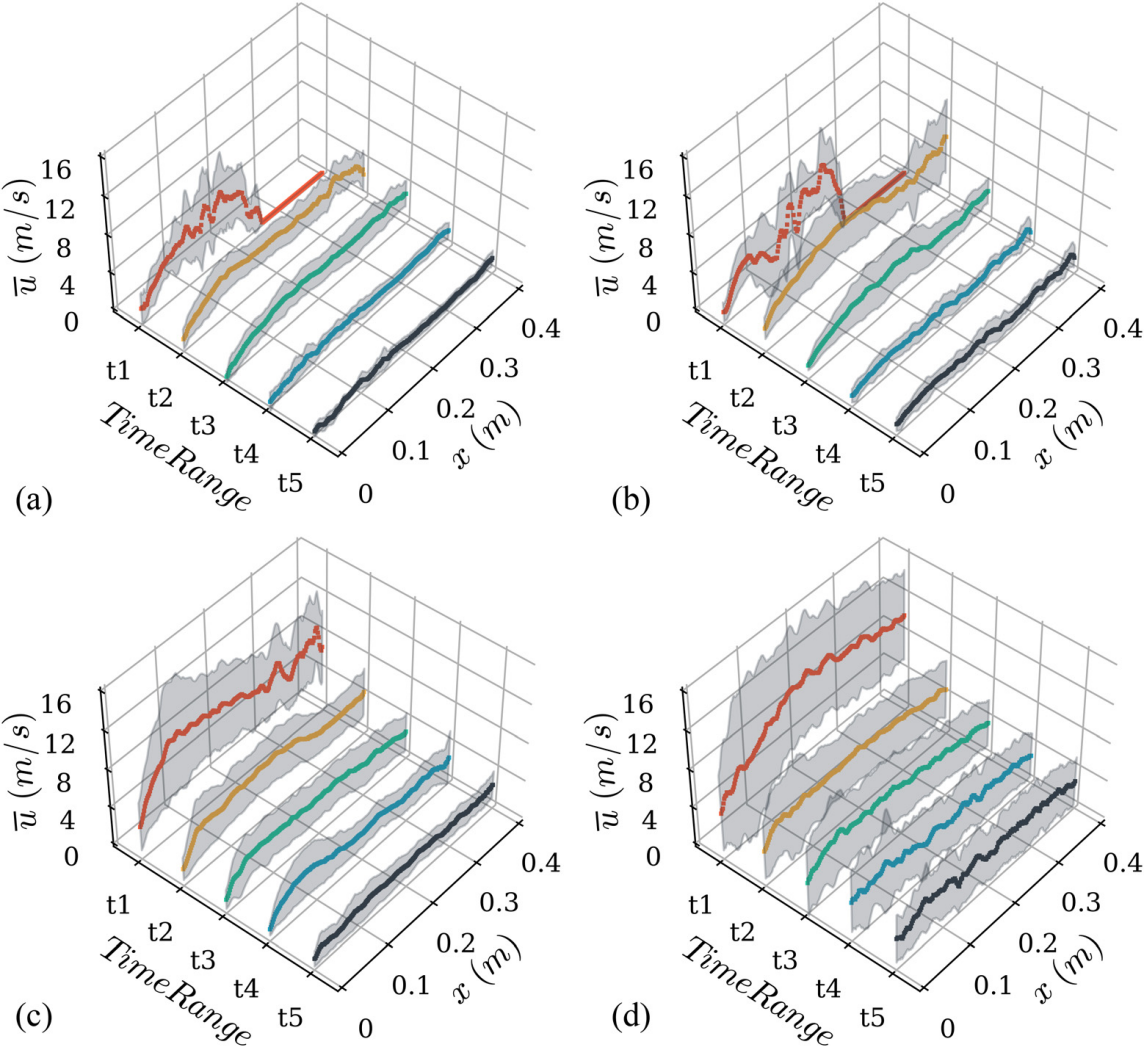
Spatial variation of mean droplet velocity for five different time-ranges (t1–t5) among the sneezes of four different subjects. Here, t1 is from 0 to 50 ms, t2 is from 50 to 100 ms, t3 is from 100 to 150 ms, t4 is from 150 to 200 ms, and t5 is from 200 to 250 ms. (a), (b), (c), and (d) are the results for subjects S1, S2, S3, and S4, respectively.

Figure 6 shows the spatial variation of the mean velocity of sneezes of all the subjects for five-time ranges, t1–t5, of 50 ms each. Time range t1 is from 0 to 50 ms, and the variation in mean velocity is highest in this range. For subjects S1 and S2, the value of mean velocity is zero for *x* > 0.28 m in t1 because the droplets have not crossed *x* = 0.28 m in the initial 50 ms. For the rest of the time ranges from t2 to t5, the droplets spanned the entire field of view for all the subjects. For time range t2 (50–100 ms), there is a significant reduction in mean velocity across the whole spatial range for subjects S3 and S4. This reduction can be explained by the peak in droplet velocities that were observed at 26 and 30 ms in Fig. 5 for subjects S3 and S4. As expected, in all the subsequent time ranges, a gradual decrease in mean velocity can be observed, and there is no appreciable change spatially across the field of view. The constant decrease in variation in mean droplet velocity from time ranges t2 to t5 implies that the droplets expelled from different sneezes of a subject exhibit self-similar motion after the loss of initial momentum. The drag force experienced by the droplets could be one of the reason for this loss of momentum after initial peak, which could also explain a more steep decline observed in mean droplet velocity as compared to airflow velocity in Fig. 4.

**FIG. 7.**
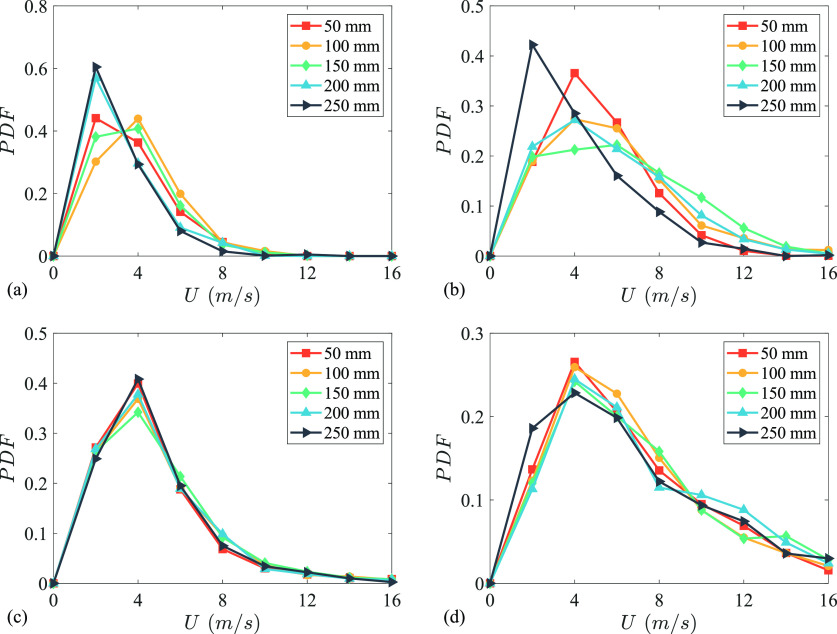
Probability density functions (PDFs) of droplet velocities at *x* = 50, 100, 150, 200, and 250 mm for (a) subject S1, (b) subject S2, (c) subject S3, and (d) subject S4.

Figure 7 shows the distribution of droplet velocities at *x* = 50, 100, 150, 200, and 250 mm for all subjects. In all the distributions, a peak was observed at approximately 4 m/s, which implies that a majority of droplets travel slower than what is usually assumed in modelling and experimental studies concerning respiratory flows.^34,35^ One of the reasons for this assumption is the lack of data on the dynamics of sneezes, which is now more readily accessible through this body of work for future studies. Additionally, the limited data from old studies focus mainly on the maximum velocity.^7,14^ For subjects S1 and S2, this peak in the distribution shifts toward lower values further downstream, implying loss of momentum of the droplets as observed in droplets tracks shown in Fig. 2. For subjects S3 and S4, the distributions were consistent across the range of *x*. At *x* = 50 mm, more than 90% of droplets have velocity less than 5.2, 7.1, 7, and 11.7 m/s for subjects S1, S2, S3, and S4, respectively. This value is 5.2 m/s (S1), 9.5 m/s (S2), 7.5 m/s (S3), and 13.2 m/s (S4) for *x* = 150 mm, and 4 m/s (S1), 6.6 m/s (S2), 7 m/s (S3), and 11.8 m/s (S4) for *x* = 250 mm. The mean, median, and standard deviations of these distributions are presented in Table I. The results reveal that the mean droplet velocity is highest in approximately *x* = 100–200 mm range, which points toward an initial increase in momentum of the droplets after the expulsion from the mouth of the subject.

**TABLE I.** Mean, median, and standard deviation (SD) of droplet velocity distributions presented in Fig. 7. All the velocities are in m/s.

	x mm	50	100	150	200	250
S1	Mean	2.6	3	2.7	2	2
	Median	2.3	2.8	2.3	1.5	1.6
	SD	1.8	1.8	1.7	1.7	1.6
S2	Mean	4	4.7	5.1	4.6	3.1
	Median	3.7	4.2	4.8	4.1	2.5
	SD	2.2	3.1	3.2	3.2	2.4
S3	Mean	3.8	3.9	4	3.7	3.7
	Median	3	3.1	3.3	3.1	3.1
	SD	3	3.2	3	2.7	2.6
S4	Mean	6	6.1	6.5	6.6	5.9
	Median	4.9	4.8	5.3	5.3	4.7
	SD	4.6	4.6	4.8	4.7	4.4

### B. Flow direction and spread

**FIG. 8.**
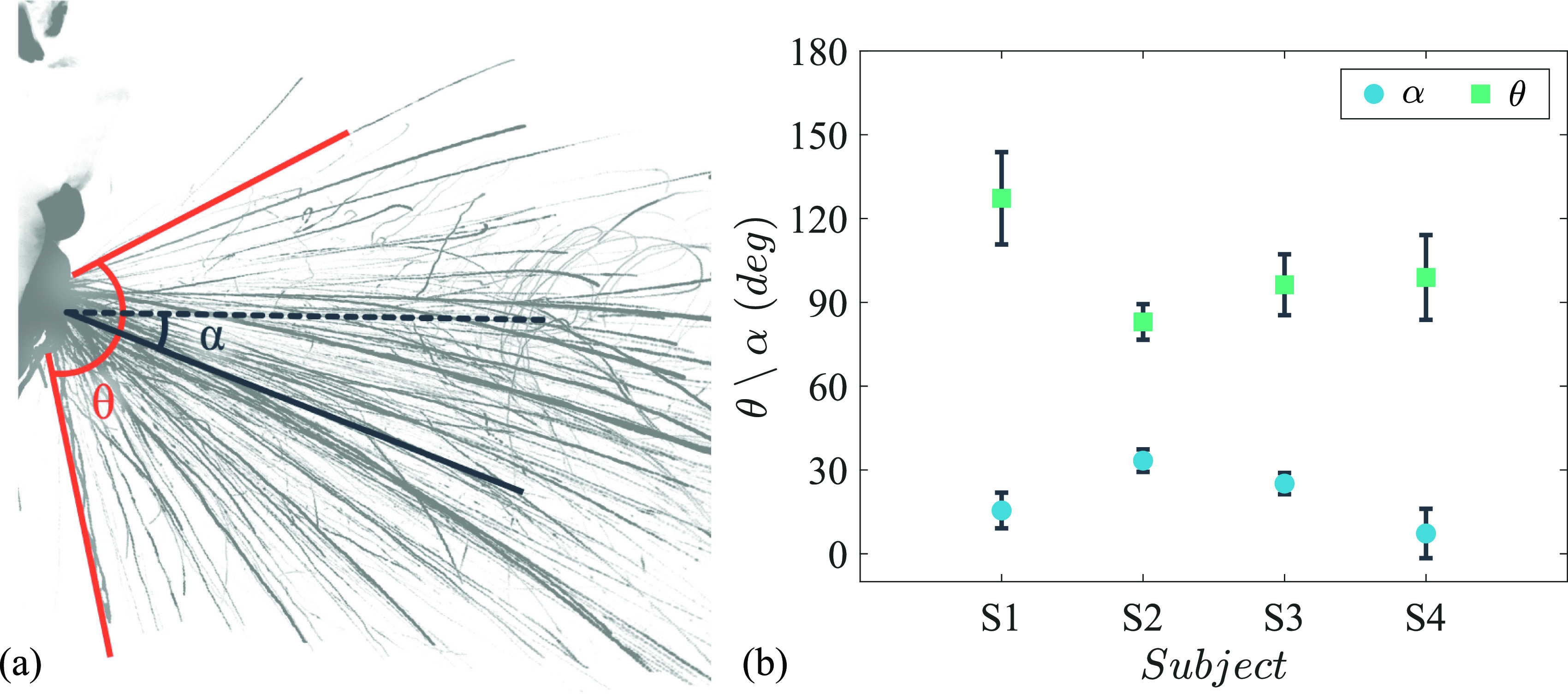
(a) Spread angle (*θ*) and flow direction (*α*) of the droplets obtained by stacking the high-speed frames of a representative sneeze. (b) Variation in angle of spread, *θ*, and flow direction, *α*, among different sneezes obtained by four volunteers.

To determine the spread of droplets expelled, *α* and *θ* are defined to find the direction of flow and the angle of spread, respectively [see Fig. 8(a)]. *θ* is the angle between the mean trajectory of the 1% topmost and 1% bottom-most droplet track, whereas *α* is the angle of the bisector of *θ* with the horizontal. The mean angle of spread was 127° (SD = 16°), 93° (SD = 6°), 96° (SD = 11°), and 11° (SD = 15°) for subjects S1, S2, S3, and S4, respectively. Other than subject S1, with an average spread angle of 127°, the spread angle among other subjects was quite consistent. Nevertheless, these values are much higher than the average spread value of 24±4° reported by Gupta *et al.*^29^ for coughs. These higher values of spread imply that the droplets expelled during a sneeze can have very different dynamics compared to a cough.

The mean direction of flow was 16° (SD = 6°), 33° (SD = 4°), 25° (SD = 4°), and 7° (SD = 9°) for subjects S1, S2, S3, and S4, respectively. For all the subjects, the flow was generally in the downward direction; however, few sneezes with the upward flow were also observed for subject S4.

### C. Head movement

**FIG. 9.**
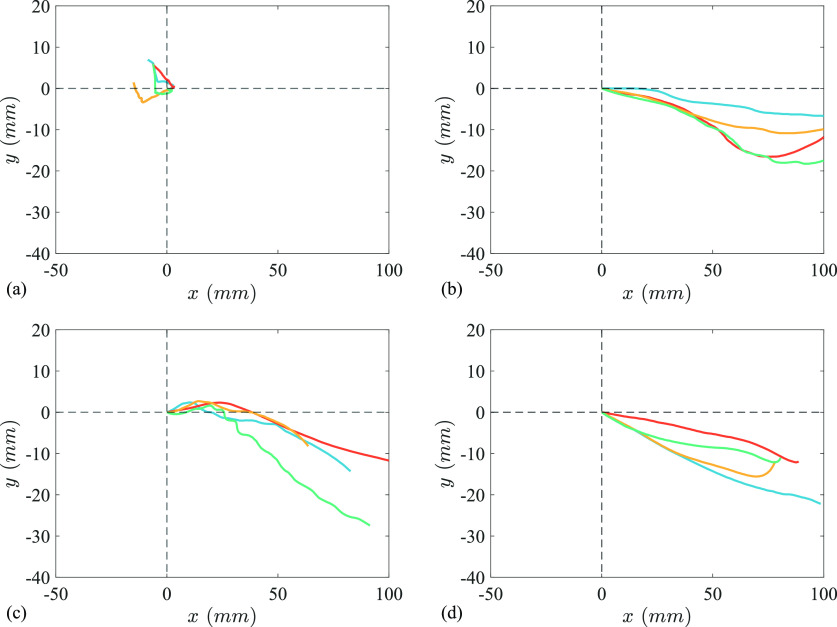
Displacement of head for four representative sneezes each from (a) subject S1, (b) subject S2, (c) subject S3, and (d) subject S4. Origin of the plot ( x=0  and y=0) represents the start of the sneeze.

**FIG. 10.**
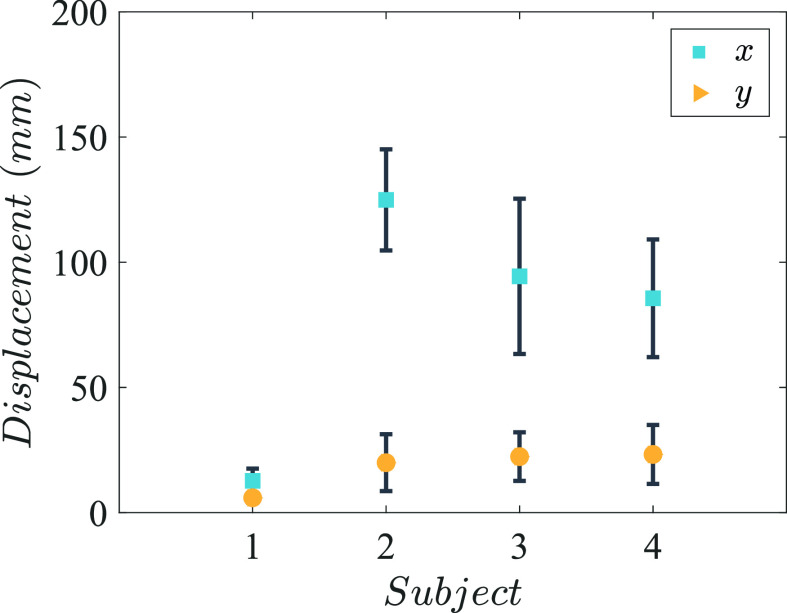
Variation in maximum horizontal (*x*) and vertical (*y*) displacements of head among all the sneezes of the four subjects.

There is a substantial movement of the head involved during sneezing, which poses an important difference to consider between subjects. Accordingly, to quantify the head movement while sneezing, the head of the subjects was tracked. This was done using a template matching algorithm to track the centroid of head in each frame. Complete details of head tracking are published in Bahl *et al.*^30^ Figure 9 shows the movement of the head for four representative sneezes from each subject. The results reveal a forward head movement of approx. 100 mm and vertical head movement of approx. 15 mm for subjects S2, S3, and S4. Conversely, the head of subject S1 moves backward during the sneeze, but the movement was within 15 mm. The average maximum displacement of head in the *x* direction was 10.9, 122.9, 93.1, and 80.6 mm, and the average maximum displacement in the *y* direction was 5.1, 19.5, 20.9, and 20.2 mm for the four subjects, respectively (see Fig. 10). The magnitude of average maximum head velocities observed among four subjects was 0.04, 0.28, 0.17, and 0.13 m/s, respectively, which were much lower than the velocities of droplets expelled from the mouth.

## IV. CONCLUSION

This study, presents the dynamics of droplets expelled during sneezing from multiple subjects, using Particle Tracking Velocimetry and light sheet illumination. The results reveal a mean droplet velocity of 2 m/s (SD = 0.65 m/s) to 5.4 m/s (SD = 0.96 m/s) across the different subjects. These values are much lower than what is usually assumed in studies simulating or replicating sneezes. Furthermore, flow direction (*α*), spread angle (*θ*), and head movement between the subjects are also quantified. The data presented will aid in improved modeling of sneezes by refining the flow characteristics of a simulated sneeze.

The results also revealed a high variation in the droplet dynamics between subjects, and even among the sneezes from the same subject, particularly for the initial 100 ms. The results of flow direction (*α*) reveal substantial variation between the subjects, from 7° to 33° in the downward direction. Similarly, a large variation in the spread angle (*θ*) and head movement was also observed between the subjects. These variations have never been accounted for in models of respiratory emissions and infection control, but it is critical to be aware of these variations. Therefore, boundary conditions for numerical simulations of sneeze flow should incorporate parameters such as flow direction, spread angle, head movement, and the associated variations for realistic understanding of the droplet spread.

## Data Availability

The data that support the findings of this study are available from the corresponding author upon reasonable request.
